# Attenuation of dengue virus infection by adeno-associated virus-mediated siRNA delivery

**DOI:** 10.1186/1479-0556-2-8

**Published:** 2004-08-09

**Authors:** Weidong Zhang, Rajeswari Singam, Gary Hellermann, Xiaoyuan Kong, Homero San Juan, Richard F Lockey, Shuen-Ju Wu, Kevin Porter, Shyam S Mohapatra

**Affiliations:** 1 Division of Allergy and Immunology-JMC Airway Disease Research Center, Department of Internal Medicine, University of South Florida; VA Hospital Tampa, FL, USA; 2 Viral Diseases Department, Naval Medical Research Center, Silver Spring, Maryland, USA

**Keywords:** dengue virus, siRNA, gene expression, adeno-associated virus

## Abstract

**Background:**

The need for safe and effective treatment of dengue virus (DEN), a class A agent that causes dengue hemorrhagic fever/dengue shock syndrome, has been a critical global priority. An effective vaccine for DEN is not yet available. In this study the possibility of attenuating DEN infection using adeno-associated virus (AAV)-encoded short interfering RNAs (siRNA) was examined in Vero cells and human dendritic cells (DCs).

**Methods:**

A cassette encoding siRNA targeted to a 3' untranslated sequence common to all DEN serotypes was designed and tested for its ability to attenuate DEN infection by use of AAV delivery.

**Results:**

Vero cells or DCs infected with AAV-siRNA showed a significant, dose-dependent reduction in DEN infection. Treatment of DCs with AAV-siRNA also decreased the DEN-induced apoptosis of DCs and did not induce significant inflammation.

**Conclusion:**

These results demonstrate that AAV-mediated siRNA delivery is capable of reducing DEN infection in cells and may be useful in decreasing DEN replication in humans.

## Introduction

The need for a safe and effective prophylaxis or treatment for dengue virus (DEN) infection, a category A mosquito-borne human pathogen, is a critical global priority. DEN causes dengue hemorrhagic fever/dengue shock syndrome (DHF/DSS), which is associated with heterologous secondary DEN infection and affects thousands of people worldwide. The incidence of DHF/DSS is increasing in the western hemisphere and, although many different approaches are being tried to develop prophylactic DEN vaccines, none have been licensed for public health and there are no specific antiviral treatments available.

DEN belongs to the family of Flaviviruses and is an enveloped single plus-stranded RNA virus with four distinct serotypes. The DEN genome of approximately 11,000 nucleotides encodes a polyprotein (C-prM-E-NS1-NS2a-NS3-NS4a-NS4b-NS5) consisting of three structural proteins (C, prM and E) and seven nonstructural proteins. The open reading frame is flanked by a 100 nucleotide-long noncoding region (NCR) at the 5' end and a 400 to 600 nucleotide-long NCR at the 3'end [1]. Although the mechanism of DEN pathogenesis is unclear, DEN typically appears to replicate locally in skin or blood dendritic cells (DCs) and may also involve monocytes and macrophages. Secondary infection is usually more serious because of antibody-dependent enhancement (ADE).

We reasoned that an effective antiviral approach aimed at attenuating the DEN virus burden might protect infected subjects from DHF/DSS and, therefore, we examined the potential of an *in vivo *gene-silencing approach using short interfering RNA (siRNA) to decrease DEN replication. RNA interference is triggered by dsRNA that is cleaved by an RNAse-III-like enzyme, *Dicer*, into 21–25 nucleotide fragments with characteristic 5' and 3' termini [2]. These siRNAs act as guides for a multi-protein complex, including a PAZ/PIWI domain containing the protein *argonaute2*, that cleaves the target mRNA [3]. These gene-silencing mechanisms are highly specific and can potentially inhibit the gene expression of different viruses [4,5]. This approach was found to be effective in blocking DEN replication in insect cells [6,7].

Plasmid DNAs or adenoviruses encoding appropriate DNA sequences allow transient siRNA expression in cells and *in vivo *leading to specific gene silencing. However, plasmids transfect mammalian cells poorly, and adenoviruses produce an acute inflammatory response and an immune response to viral vector-encoded antigens [7,8]. We therefore developed an adeno-associated virus (AAV) system capable of expressing siRNA cassettes, and tested this vector with a siRNA cassette composed of a nucleotide sequence from the 3' NCR of the DEN genome (siDEN3UT), which is common to all four serotypes. The results obtained in Vero cells and human DCs infected with AAV-siDEN3UT show significant decreases in DEN infection and DEN-induced apoptosis.

## Methods

### Plasmid constructs

The pCMV-MCS plasmid (Stratagene) was digested with *Not *I and the larger fragment was ligated to the synthetic adapter containing in order, *Not *I-*Kpn *I-*Apa *I-*Xho *I-*Hind *III-*EcoR *I-*Bam *HI-*Sac *II-*Sac *I-*Cla *I-*Sal *I-*Bgl *II-*Not *I. The U6 promoter was obtained by PCR amplification, using specific primers with the desired restriction sites from the template pSilencer 1.0-U6 (Ambion), and inserted into the adaptor at the *Kpn *I and *Apa *I sites to get a novel plasmid pCMV-U6.

Pairs of oligos were synthesized to develop siRNA constructs. The nucleotide sequence for each siRNA is as follows: siEGFP: 5'-GGC GAT GCC ACC TAC GGC AAG CTT CTC GAT TCG AAG CTT GCC GTA GGT GGC ATC GCC CTT TTT G-3' [10]; siRSVNS1:5'-GGC AGC AAT TCA TTG AGT ATG CTT CTC GAA ATA AGC ATA CTC AAT GAA TTG CTG CCT TTT TG-3'; siDENPrM: 5'-GGA AGA CAT AGA TTG TT G GTG CAC TCG AGT CAA CGT GCA CCA ACA ATC TAT GTC T TC CCT TTT TG-3'; siDEN3UT:5'-GGA AAA ACA GCA TAT TGA CGC TGC TCG AGT CAA CGC AGC GTC AAT ATG CTG TTT TTC CCT TTT TG-3'. Each pair of oligos was annealed and then inserted into pCMV-U6 digested with *Apa *I/*Xho *I and *Xho *I/*EcoR *I respectively. The modified pCMV-U6 plasmid was then redigested with *Not *I and the smaller fragment was ligated to the 2.9 kb fragment of pAAV-MCS (Stratagene) obtained following its *Not *I digestion to generate the corresponding si-vector for EGFP, RSV and DEN. HEK-293 cells were cotransfected with the helper plasmid and the si- plasmid to generate recombinant AAV.

### Cell culture and viral packaging

HEK293 cells were cultured with DMEM (Cellgro) plus 10% FBS (Cellgro) and cotransfected with pSMWZ-siDEN, pHelper and pAAV-RC (Stratagene) by standard calcium phosphate transfection. Cells were harvested 48 hr post-transfection and the cell pellets were stored at -80°C.

### Purification of recombinant adenoassociated viruses

Cells were lysed by 5 cycles of freezing and thawing to release the virus. Crude viral lysate were collected by centrifugation at 27,000 × g for 30 min, and the supernatants were harvested and put onto a CsCl gradient (density 1.20/1.50) in fresh tubes and centrifuged for 16 h at 100,000 × g. Opalescent bands were collected after ultracentrifugation. Titers of purified AAVs were measured using an AAV titration ELISA kit (Progen Biotechnik, Germany).

### Isolation and culture of dendritic cells from human peripheral blood

Conditions were similar to those described previously [11]. Buffy coats were diluted with one volume of DMEM (Cellgro) and PBMCs were isolated by density-gradient centrifugation using Histopaque-1077 (SIGMA) according to the instructions. The PBMC layer was harvested, washed twice with DMEM, resuspended in DMEM supplemented with 10% FBS, and then seeded into six-well culture plates. After 2 h at 37°C/5%CO_2 _the nonadherent cells were removed and the adherent cells were cultured with fresh DMEM supplemented with 10% FBS (Cellgro), 200 ng/ml IL-4 (BD-Pharmingen) and 50 ng/ml GM-CSF (BD-Pharmingen) for 7 days prior to infection with DEN.

### Blocking dengue virus infection in vitro

1 × 10^5 ^Vero cells or DCs were seeded into six-well tissue culture plates and infected with different numbers of recombinant AAV carrying the DEN-siRNA silencing cassette. After 2 days the cells were infected with DEN-2 virus (strain16803) at an MOI of 0.1.

### Flow cytometry

Cells were harvested and centrifuged for 10 min at 150 × g. The cell pellets were washed with PBS and resuspended with antibody to DEN-2 virus envelope protein, (Microbix Biosystems Inc, Clone No 3H5) on ice for 40 min. The cells were centrifuged and pellets were washed with PBS and then resuspended with secondary antibody conjugated with FITC (Sigma) for an additional 30 min. The number of infected cells was measured by flow cytometry 5 days post-infection. DCs were also stained with CD11c antibody conjugated with PE (BD-Pharmingen).

### Plaque assay

The supernatants from DEN-2-infected DCs were collected at day 5 post-infection and 10-fold serial dilutions were allowed to adsorb to monolayers of Vero cells in six-well culture plates for 2 h. The medium was then removed and replaced by an agarose-containing overlay (2X DMEM, 10%FBS, non-essential amino acids (Gibco BRL), 1% low melting-point agarose (Gibco BRL) and the plates were incubated at 37°C/5% CO_2 _for 5 days. Afterwards, 1% neutral red (Sigma) was added to each well and the plaques were counted 48 h later.

### Apoptosis assay

Infected-DCs were harvested on day 5 of infection with DEN-2 virus. Aliquots of DCs were put onto slides using a Cytospin and fixed with 4% paraformaldehyde. Apoptotic DCs were determined using the terminal dUTP nick end-labeling assay (TUNEL, Promega, Madison WI) and the annexin V apoptosis detection kit (BD Biosciences, CA).

### Cytometric bead array and ELISA analysis

Supernatants from infected-DCs were harvested at 24 h, 48 h, 72 h and 96 h post-infection. Cytokine concentrations were measured by cytometric bead array (CBA) and ELISA (BD-Pharmingen) following instructions in the manuals.

### Statistical analysis

Data were expressed as arithmetic mean ± SEM. Levels of significance of the differences between groups were determined by the student *t *test. Values of *p *< 0.05 were considered statistically significant.

## Results

### Development of an AAV-siRNA system for gene silencing

**Figure 1:**
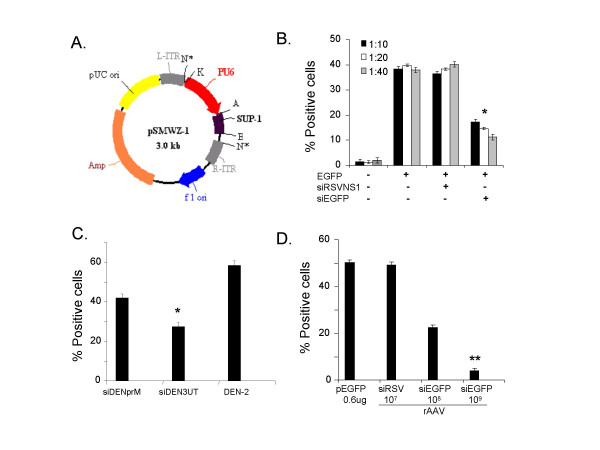
Construction and characterization of the siDEN suppressor. **(A) **Diagram of the construction of the plasmid vector, pSMWZ-1, capable of expressing a DEN infection suppressor cassette. Abbreviations: N*, Not I; K, Kpn I; A, Apa I; E, EcoR I; SUP-1, Suppressor cassette; **(B) **Co-transfection with pSMWZ-siEGFP and pEGFP inhibits the expression of EGFP in cultured cells. HEK293 cells were transfected with different concentrations of plasmid DNA and three days later, EGFP-positive cells were counted by fluorescence microscopy. Results are expressed as mean ± SEM. *p < 0.05 compared to control (siRSVNS1, an unrelated siRNA construct against respiratory syncytial virus). **(C) **pSMWZ-siDEN suppression of DEN-2 virus replication. Vero cells were transfected with pSMWZ-siDEN3UT or pSMWZ-siDENpreM plasmid. Two days later, the cells were infected with DEN-2 virus (MOI of 0.1) and 5 days later, the numbers of DEN-2 virus infected cells were counted by fluorescence microscopy. Data are mean ± SEM. *p < 0.05 compared to control DEN-2. **(D) **AAV-siEGFP inhibits the expression of EGFP in cultured cells. HEK293 cells were infected with different concentrations of AAV-siEGFP, and three days later the cells were transfected with pEGFP. EGFP-positive cells were counted by fluorescence microscopy. Statistically significant differences, **p < 0.01, when compared to pEGFP plasmid control, AAV-siRSV (10^7^) and AAV-siEGFP (10^8^) group, respectively.

In order to develop an AAV-siRNA system, a plasmid pSMWZ-1 was engineered that comprised a mouse U6 promoter linked to a siRNA cassette (Fig. 1A). To test whether this plasmid was functional and capable of suppressing gene expression, HEK293 cells were cotransfected with pEGFP, a plasmid expressing green fluorescent protein, and pSMWZ-siEGFP. The percentage of cells expressing EGFP was determined and the results showed that there was a dose-dependent silencing of EGFP expression (Fig. 1B). In contrast, control cells cotransfected with siRSVNS1 (targets the NS1 gene of human respiratory syncytial virus) in place of siEGFP did not show any reduction in EGFP expression. To test various siDEN candidates, Vero cells were transfected with either pSMWZ-siDENpreM (siDENpreM) or pSMWZ-siDEN3UT(siDEN3UT), then two days post-transfection infected with DEN-2 (strain 16803) at an MOI of 0.1. At five days post-infection, the numbers of DEN-2 virus-infected cells were quantified by fluorescence microscopy using antibody to DEN-2 envelope protein and FITC-conjugated secondary antibody. The results showed that siDEN3UT was better than siDENpreM in suppression of DEN-2 infection (Fig. 1C). The AAV-siRNA system was similarly tested using HEK293 cells which were infected with AAV-siEGFP and then transfected with pEGFP. The decrease in the percentage of cells expressing EGFP showed that there was a silencing of EGFP expression in a dose-dependent and sequence-specific manner (Fig. 1D).

**Figure 2:**
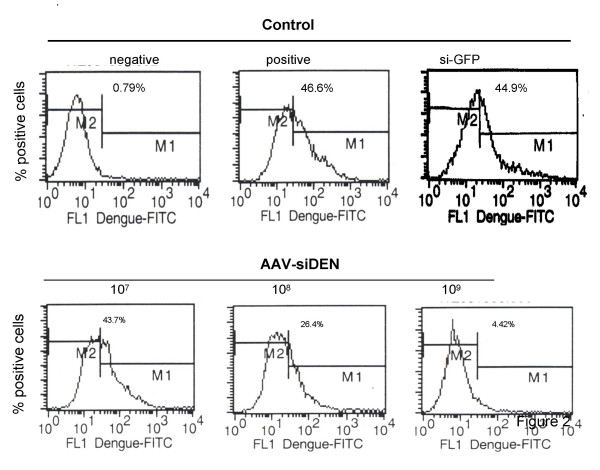
AAVsiDEN expression decreases DEN-2 virus infection in cultured Vero cells. Cells were infected with different amounts (PFU/ml) of AAV carrying the siDEN3UT silencing cassette and after 2 days the cells were infected with DEN-2 virus (MOI of 0.1). Five days later, the numbers of DEN-2 virus infected cells were measured by flow cytometry.

To test whether AAV-siDEN-2 expression decreases DEN-2 virus infection in cultured Vero cells, cells were infected with recombinant AAV carrying siDEN-2 (MOI 10) or siEGFP (MOI ~1000) silencing cassettes. After 2 days the cells were infected with DEN-2 virus at an MOI of 0.1. Five days later, the numbers of DEN-2 virus infected cells were quantified by flow cytometry using anti-DEN-envelope protein. Cells pre-infected with AAV-siDEN3UT, but not AAV-siEGFP, showed a significant reduction in DEN infection, and the reduction was dose dependent (Fig. 2).

### siRNA suppresses DEN infection in human dendritic cells

**Figure 3:**
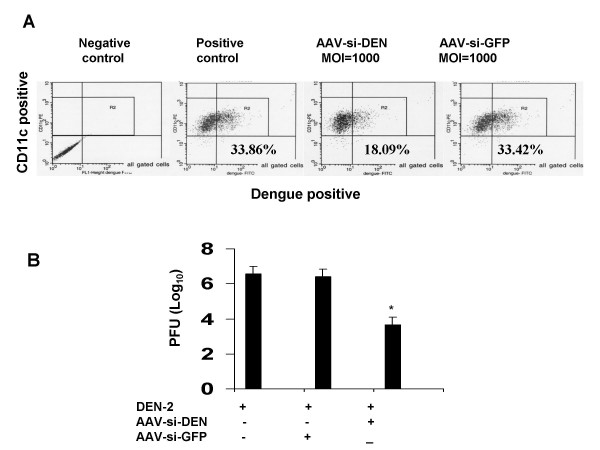
Suppression of DEN-2 replication in DCs by siDEN. **(A) **DCs were isolated from human peripheral blood and cultured in DMEM medium supplemented with FBS, IL-4 and GM-CSF. Non-adherent DCs were harvested on day 7, infected with AAVsiDEN, and two days later the cells were infected with DEN-2 at 0.1 MOI. DCs were harvested 5 days after DEN-2 infection and DEN-2 titers were measured by flow cytometry. **(B) **Supernatants from DEN-infected DCs were collected and added to culture plates containing confluent Vero cell monolayers. After virus adsorption, the Vero cells were overlaid with agarose and stained with 1% neutral red. Viral plaques were counted 48 h after neutral red overlay. Data are the averages of two independent experiments. *p < 0.05 compared to control.

DEN is transmitted through *Aedes aegypti *mosquito bites, and resident skin DCs are regarded as the targets of DEN infection [12]. DCs are thought to be 10-fold more permissive for DEN infection than monocytes or macrophages [13]. We therefore tested the ability of AAV-siDEN3UT to attenuate DEN infection in human DCs. DCs were isolated from human blood and cultured in the presence of IL-4 and GM-CSF for 5 days to generate immature DCs (iDCs). These DCs were then infected with 10^9 ^PFU/ml of recombinant AAV carrying siDEN3UT or siEGFP (control) silencing cassette. After 2 days the cells were infected with DEN-2 at an MOI of 0.1. Five days later, the numbers of DEN-2 infected cells were quantified by flow cytometry using DEN-2 antibody. Cells preinfected with AAVsiDEN3UT showed a 50% reduction in the number of infected cells (Fig. 3A). To test whether the reduction in the number of infected DCs involved a reduction in DEN titer, the culture supernatants were examined using a Vero cell-based plaque assay. AAV-mediated siDEN3UT expression significantly decreased DEN-2 virus titer compared to control (Fig. 3B). These results indicate that AAV-siDEN3UT can significantly decrease DEN titers in human DCs.

### Silencing DEN-2 genes inhibits apoptosis in dendritic cells

**Figure 4:**
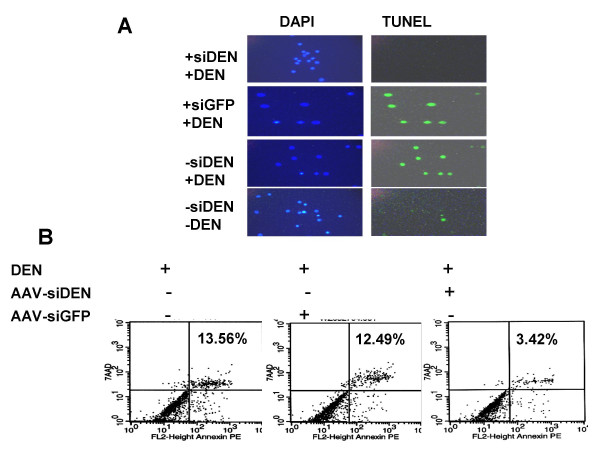
AAVsiDEN reduces apoptosis in human DCs infected with DEN-2. DCs were isolated from human peripheral blood and infected with AAVsiDEN followed by DEN-2. Five days after infection, DCs were put onto slides and apoptosis was determined using the terminal dUTP nick end-labeling assay (TUNEL). Nuclei were stained with diamidinophenylindole (DAPI). Representative fields were visualized by fluorescence microscopy.

It has been reported that DEN infection induces apoptosis of DCs [11] leading to an immunosuppressed condition. To examine the effect of AAV-mediated siRNA delivery in DCs, apoptosis was investigated in infected DCs using the TUNEL assay. The results showed that a small percentage of DCs undergo apoptosis naturally during culture, but DEN infection causes much more apoptosis. The AAV-siRNA-treated DEN-infected DCs showed significantly fewer apoptotic cells compared to DEN-infected cells without AAV-siRNA (Fig. 4).

### Differential expression of cytokines by infected dendritic cells

**Figure 5:**
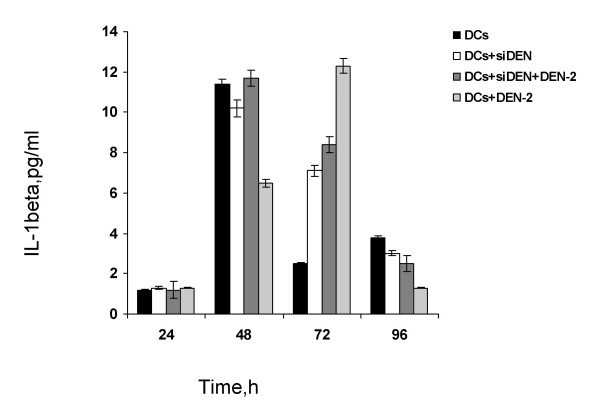
Differential expression of cytokines in the supernatants of infected DCs. Supernatants from DEN-2-infected DCs with or without siDEN treatment were harvested at the indicated time points and analyzed by CBA and ELISA to measure the concentrations of cytokines. Data are the averages of two independent experiments. **p < 0.01 in comparison with the value of DCs within individual group.

The supernatants of infected DCs were collected at different time points and cytokines were measured using cytokine bead array (CBA) and ELISA assays. As showed in Figure 5, cultured DCs spontaneously produced increased IL-1b. A variety of cytokines including IFN-γ, TNF-α, IL-8, IL-6, IL-12 were measured (data not shown). In the presence of AAV-siRNA infection, the production of IFN-γ, TNF-α, IL-8, IL-6, and IL-12 did not change significantly compared with cultured DCs. IL-1b secretion at 72 h post-infection was increased, however. These results indicate that in our system, AAV-siRNA delivery does not induce acute inflammation in DCs, in vitro,

## Discussion

The significant findings of this report include the development of an adeno-associated virus-based siRNA approach for downregulating gene expression. A recombinant AAV-siDEN3UT was utilized to induce significant decreases in DEN infection compared to control in both Vero cells and human DCs. The results indicate that siRNAs may be used to attenuate DEN infection in human DCs and may have therapeutic value.

Interference of gene expression by siRNAs is a novel strategy to knock down specific genes in cells or tissues, and the specific silencing of pathogen genes using siRNA is a very attractive approach for the clinical treatment of infectious diseases. Long dsRNAs (of >30 nt in length) activate a dsRNA-dependent protein kinase and 2', 5'-oligoadenylate synthetase in mammalian cells, which leads to a non-specific reduction in levels of mRNAs [14]. The endogenous expression of siRNAs from introduced DNA templates is thought to overcome some limitations of exogenous siRNA delivery, in particular its transient effects on silencing specific genes and loss of phenotype [15]. AAV vectors have been proven to be safe and efficacious in Phase I clinical trials for gene therapy of cystic fibrosis and hemophilia B and are regarded as a potential alternative to retroviral and adenoviral vectors for gene therapy in humans. The AAV vectors have a number of advantages over other vectors. They are not pathogenic and do not induce production of neutralizing antibodies that could reduce transgene function. They possess a broad-range of tissue tropism and the capability of inducing long-term transgene expression [16]. In this study, we utilized a novel AAV system to deliver DEN siRNA into mammalian cells and estimated its anti-DEN effect *in vitro. *In this AAV system, we incorporated the mouse U6 promoter, which is important for transcription and folding of the suppressor RNA, into a plasmid pCMV-U6.

The choice of appropriate target genes is necessary for the success of the siRNA strategy, and two siRNAs derived from either the pre-M or the 3' NCR region of DEN-2 were used in our study. An internal deletion of 3' NCR nucleotide sequences was found to be lethal for DEN virus replication in an *in vivo *study [17]. The 3' NCR of the flavivirus genome, which presumably functions as a promoter for minus-strand RNA synthesis, is predicted to form a stem-and-loop secondary structure. Computational analyses have revealed that there is conserved sequence in all flaviviruses within the 3' end [18,19]. Thus, two siRNA cassettes were tested in this study that included the 3'NCR sequence common to all four DEN serotypes. The other siRNA cassette is from the gene encoding the preM protein which is important for maturation of the virus into an infectious form. Our test of anti-DEN efficiency showed that siDEN3UT attenuated DEN Infection better than siDENpreM. Knocking down viral genes at the earlier stage of the viral multiplication cycle rather than in the structural protein synthesis phase may provide better antiviral protection, although the limited plasmid transfection ratio appeared to influence the suppression efficiency of siDEN to DEN-2 infection in Vero cells in the present study (Fig. 1C). The other DEN serotypes will be investigated with our 3'NCR cassette.

DEN is transmitted through *Aedes aegypti *mosquito bite, and resident skin DCs are an early target of DEN infection [12]. Immature DCs are the most permissive for DEN infection and serve as a source of DEN replication and production [20]. Replication in the early target cells may be essential for dengue pathogenesis in the human host. In this study, we also utilized peripheral blood iDCs as a cell model to test our AAV system. Similar to results in Vero cells, AAV-mediated siDEN3UT delivery down-regulated DEN-2 protein expression in iDCs. However, the magnitude of suppression in iDCs at the same infectious titer of AAV-siDEN was less compared to that found in Vero cells. Previous data showed that variations in the efficiency of transduction among DCs derived from different normal blood donors is between 2% and 50% [21], and we found that the infectious ratio for AAVEGFP is about 45%~50% in Vero cells. That may be due to limited expression of the AAV receptor or differential activation of the mouse U6 promoter in Vero cells compared to DCs [22]. Increasing the AAV infection titer or utilizing a more effective promoter within the AAV vector backbone might elevate the suppression for DEN replication in iDCs. Nevertheless, DCs treated with recombinant AAV showed a significant reduction in DEN virus titer compared to control. This is important as viral titer is the gold standard for measuring antiviral activity.

DCs are one of the most powerful of APCs. After infection with virus in the periphery, iDCs process viral antigens, then differentiate into mature DCs and migrate from peripheral tissues to lymph nodes where they prime naïve CD4 and CD8 T lymphocytes to maintain protective antiviral cytotoxic T cell memory [23,24]. Thus, DCs play an important role in the initiation of antiviral immunity and provide a crucial step in the development of adaptive antiviral immunity. Previous data showed that DEN infection induces apoptosis of DCs [11], which leads to a state of temporary immune-suppression during DEN fever. An important observation in our study is that AAV-siDEN treatment resulted in a significant decrease in apoptotic iDCs. The attenuation of apoptosis in iDCs following AAV-mediated siRNA delivery suggests that AAV-siRNA may be immunologically protective. After the primary DEN infection, most patients appear viremic in the early febrile phase, but the viruses are quickly cleared from the blood system after defervescence [25]. The activation of both a humoral and cellular immune response is considered to be involved in DEN clearance. The most severe outcome in DEN infection is development of DHF/DSS, which is associated with secondary infections by heterotypic DEN serotypes. It is postulated that the preexisting, cross-reactive, adaptive immune response leads to excessive cytokine production, complement activation, and the release of other inflammatory factors that produce DHF/DSS [20]. Therefore, it should be imperative for prophylaxis of DHF/DSS to eliminate DEN infection by different serotypes in the early target cells. Attenuation of DEN infection in DCs and protection of infected DCs from apoptosis would be a benefit for the elimination of the early DEN infection and the development and maintenance of antiviral innate/adaptive immune response *in vivo*.

One of the important features of AAV vectors is the lack of inflammation following infection. We failed to detect significant IFNγ or IL-12 production in the supernatants of AAV-siDEN-infected DCs. This is in accordance with previous data [26-28], which demonstrated our AAV delivery system did not induce significant acute inflammatory responses and, therefore, is useful in gene therapy for DEN infection in humans.

In conclusion, we developed a novel AAV-mediated siRNA delivery system. Our results demonstrate significant downregulation of DEN protein expression in Vero cells and human DCs, which strongly suggest that our AAV vector can be useful for siRNA delivery and that this AAV system may be applied in clinical settings to attenuate DEN infection.

## List of abbreviations

AAV, adeno-associated virus; DCs, dendritic cells; DEN, dengue virus; DHF/DSS, dengue hemorrhagic fever/dengue shock syndrome; MOI, multiplicity of infection; pEGFP, enhanced green fluorescent protein; siRNA, small interfering RNA; TUNEL, terminal deoxynucleotidyl transferase-mediated dUTP nick end-labeling.

## Competing interests

None declared.

## Authors' contributions

WDZ constructed the si-plasmids, performed cell culture, isolated and administered the virus and isolated and cultured dendritic cells. RS assisted with virus handling and cell culture, and performed flow cytometry. GH did TUNEL assays. XK did cytometric bead array assays and ELISAs. HSJ measured virus titer by plaque assay. RFL, SW, KP and SSM designed and implemented the experiments, performed troubleshooting, and did the analysis and interpretation of the data.
